# Development, validation, and visualization of a web-based nomogram for predicting chronic kidney disease incidence at health examination centers

**DOI:** 10.1080/0886022X.2024.2398183

**Published:** 2024-10-08

**Authors:** Chuxuan Luo, Lanjun Fu, Lin Liu, Maosheng Chen, Kunliang Chen, Yiwen Li, Bo Lin, Juan Jin, Bin Zhu, Qiang He, Lina Shao

**Affiliations:** a Department of Nephrology, Taizhou Hospital of Zhejiang Province, Wenzhou Medical University, Taizhou, Zhejiang, China; b Department of Nephrology, the First Affiliated Hospital of Zhejiang Chinese Medical University (Zhejiang Provincial Hospital of Traditional Chinese Medicine), Hangzhou, Zhejiang, China; c Urology & Nephrology Center, Department of Nephrology, Zhejiang Provincial People’s Hospital, Affiliated People’s Hospital, Hangzhou Medical College, Hangzhou, Zhejiang, China; d Center for General Practice Medicine, Zhejiang Provincial People’s Hospital, Affiliated People’s Hospital, Hangzhou Medical College, Hangzhou, Zhejiang, China

**Keywords:** Chronic kidney disease, nomogram, health examination, early detection, prediction model

## Abstract

**Purpose:**

To develop and validate a web-based nomogram for predicting new incident chronic kidney disease (CKD) within 4 years in a cohort undergoing routine physical examination from two health examination centers.

**Methods:**

One center was utilized for training and internal validation of a nomogram model involving 6515 patients, while a separate center was employed for external validation with 3152 patients. Sixteen candidate predictors, including patient demographics, medical histories, physical examination, and laboratory test data, were included in this study to ascertain factors linked to new incident CKD. A nomogram was created to predict CKD risks using a logistic model. The nomogram’s performance was assessed using the area under the receiver operating characteristic curve (AUC), calibration plot, and decision curve analysis.

**Results:**

Out of the 9667 healthy individuals included in the study with mean age of 46 years, sex ratio (male/female) of 1.69 (6075/3592), 118 (2.59%), 51 (2.61%), and 60 (1.90%) individuals developed CKD in the training (*n* = 4563), internal validation (*n* = 1952), and external validation (*n* = 3152) datasets, respectively. Age, history of diabetes mellitus, systolic blood pressure, serum creatinine, albumin, and triglyceride levels were used to build the nomogram, which yielded excellent discrimination ability (training cohort, AUC = 0.8806, 95% confidence interval [CI] 0.8472–0.9141; internal validation cohort, AUC = 0.8506, 95% CI 0.7856–0.9156; external validation cohort, AUC = 0.9183, 95% CI 0.8698–0.9669). We further developed a web-based calculator for convenient application (https://luochuxuan.shinyapps.io/dynnomapp/).

**Conclusion:**

Our web-based nomogram accurately predicted CKD risks in Chinese health individuals and can be easily used in clinical settings.

## Introduction

Since the release of the Kidney Disease Outcomes Quality Initiative (K/DOQI) guidelines in 2002, chronic kidney disease (CKD) has been considered a significant global public health issue [1,2]. According to the latest epidemiological data, 8.2% of the adult population in China has CKD [3]. Owing to the insidious onset and atypical symptoms of CKD, it progresses to end-stage kidney disease in many patients, and the affected patients miss optimal treatment opportunities before receiving a final diagnosis. Between 4.9–7.1 million patients worldwide require renal replacement therapy [4]. In China, the annual cost of dialysis accounts for 10% of the total health expenditure [5]. Hence, identifying individuals with a heightened susceptibility to CKD at an early stage is important.

Over the past few decades, the prevailing medical practice model has transitioned from experience-based medicine to evidence-based medicine, followed by precision medicine [6]. In modern medicine, individualized treatment strategies and risk predictions are of increasing significance. To simultaneously avoid excessive medical treatments for low-risk individuals and justify preventive interventions for high-risk individuals, accurate predictive tools are urgently required in clinical practice. Although numerous risk prediction models for CKD are available, many aspects of these models need to be improved, such as methodological flaws and non-standardized reporting of outcomes [7]. This leads to poor performance when assessed using new datasets. The nomogram, a multifactor calibrated visualization tool, has been extensively utilized in clinical practice for predicting a variety of outcomes. It provides clinicians with a foundation for developing more effective and individualized treatment plans. Therefore, we have developed and validated a novel web-based nomogram to predict new incident CKD within 4 years, utilizing validated methods in a cohort undergoing routine physical examinations.

## Methods

### Study design and population

This was a retrospective cohort study. A total of 35,623 participants undergoing annual routine physical examinations at two Chinese university hospitals (Zhejiang Provincial People’s Hospital and Zhejiang Provincial Hospital of Traditional Chinese Medicine) between January and December 2017 were consecutively recruited. The inclusion criteria were as follows: 1) age 18–75 years and 2) participation in annual physical examinations for five consecutive years in the same hospital. The exclusion criteria were as follows: 1) previous history of CKD; 2) severe medical conditions (cardiovascular and cerebrovascular diseases, liver and kidney dysfunction, and heart failure); and 3) missing values for serum creatinine at baseline. Based on the aforementioned criteria, 25,956 individuals were excluded (1134 because of missing values for serum creatinine at baseline, 703 because of having an estimated glomerular filtration rate [eGFR] less than 60 mL/min/1.73 m^2^ at baseline, 703 because of age less than 18 years or greater than 75 years, and 23,416 for having a follow-up time of less than four years). This study ultimately included 9667 participants who satisfied all inclusion criteria. Among them, 6515 individuals from Zhejiang Provincial People’s Hospital were randomly allocated to the training (*n* = 4563) and internal validation (*n* = 1952) datasets at a ratio of 7:3. The remaining 3152 individuals from the Zhejiang Provincial Hospital of Traditional Chinese Medicine were designated as the external validation cohort. A flowchart depicting the study is shown in Appendix 1, supplementary material.

### Data collection

Data on demographics (e.g., age, sex, and body mass index), medical histories (e.g., history of hypertension or diabetes mellitus (DM)), physical examination findings (e.g., systolic and diastolic blood pressure), and laboratory test results (e.g., albumin, serum creatinine, uric acid, triglyceride, high-density lipoprotein cholesterol, low-density lipoprotein cholesterol, glycated hemoglobin A1c [HbA1c], and hemoglobin) at baseline and during follow-up were retrospectively collected from the hospitals’ electronic data management systems.

### Ethical approval and informed consent

This study was approved by the Ethics Committees of Zhejiang Provincial People’s Hospital (reference number: 2019KY277) and Zhejiang Provincial Hospital of Traditional Chinese Medicine (reference number: 2022-KL-160-01). All procedures conducted in this study were formulated and performed in compliance with the guidelines outlined in the Declaration of Helsinki (revised in 2013) [8]. As this was a retrospective study, informed consent was not required.

### Definitions

The eGFR was calculated using the 2009 Chronic Kidney Disease Epidemiology Collaboration (CKD-EPI) formula [9], and CKD was defined as having an eGFR <60 mL/min/1.73 m^2^ for 1 or more consecutive years, in accordance with existing literature [10]. Participants were followed up for 4 years, with the development of CKD being the endpoint event. Hypertension was defined according to self-reported history and/or a blood pressure measurement of ≥140/90 mmHg, while DM was defined according to self-reported history and/or a HbA1c level of ≥6.5%.

### Statistical analyses

In the statistical analyses, when the proportion of missing values for a variable was less than 20%, the missing variable was handled using multiple imputations in SPSS (version 25.0, IBM), following the assumption that data were missing completely at random, such that the variables observed and recorded were predictive of the missing data.

All collected data were entered into Microsoft Excel 2007 and transferred to the R software (version 4.1.2) for statistical analysis. The R package ‘RMS’ was employed to randomly divide individuals into the training dataset for establishing the nomogram and the internal validation dataset for assessing nomogram performance at a ratio of 7:3. Given that the outcome variable of interest was a binary variable (i.e., the development of CKD within a 4-year period), univariate and multivariate logistic regression analyses were sequentially conducted to determine independent predictors of CKD.

The normality of the distribution for each continuous variable was assessed using the Kolmogorov–Smirnov test. All continuous variables were found to be non-normally distributed and are presented as medians and interquartile ranges. Differences between groups were analyzed using two independent sample non-parametric tests. Categorical variables, compared using the chi-squared or Fisher’s exact tests, are presented as frequencies (percentages). All significant variables identified by univariate analysis were included in a multivariate logistic analysis after assessing for multicollinearity. The ‘RMS’ and ‘DynNom’ packages in R software were used to construct both a conventional nomogram and a web-based nomogram, selecting variables through a backward stepwise selection based on the Akaike information criterion (AIC), with preference for models with smaller AIC values indicating better fit.

Additionally, the nomogram performance was evaluated by assessing its discrimination, calibration, and clinical utility using the receiver operating characteristic area under the curve (AUC), calibration plot, and decision curve analysis (DCA). The AUC and discrimination were categorized as outstanding (0.9, 1), excellent (0.8, 0.9), acceptable (0.7, 0.8), poor (0.6, 0.7), or failed (0.5, 0.6) [11]. DCA is a novel approach that has been introduced for assessing the clinical effectiveness of nomograms by illustrating the overall benefit of various decision strategies across different probability thresholds [12]. Statistical significance was determined by a two-sided p-value less than 0.05 for all statistical analyses.

## Results

### Baseline characteristics of the training and internal validation datasets

Among the 9667 healthy individuals with mean age of 46 years, sex ratio (male/female) of 1.69 (6075/3592), 118 (2.59%), 51 (2.61%), and 60 (1.90%) developed CKD in the training (*n* = 4563), internal validation (*n* = 1952), and external validation (*n* = 3152) datasets, respectively. The duration of time to develop CKD ranged from 1 year to 4 years, in which 1 year, 2 years, 3 years and 4 years accounted for 21.19% (25 cases), 21.19% (25 cases), 21.19% (25 cases), and 36.43% (43 cases) in the training, 35.29% (18 cases), 9.80% (5 cases), 21.57% (11 cases), and 33.34% (17 cases) in the internal validation, and 6.67% (4 cases), 5.00% (3 cases), 73.33% (44 cases), and 15% (9 cases) external validation datasets, respectively. The pairwise comparisons of baseline clinical features were made among the training, internal validation and external validation datasets, and there were no statistically significant differences in clinical characteristics between the training and internal validation cohorts (all *p* > 0.05). The findings are presented in Table 1, Appendices 2 and 3, supplementary material.

**Table 1.** Comparison of baseline characteristics between training cohort and internal validation cohort.

Variables		Training cohort (*n* = 4563)	Internal validation cohort (*n* = 1952)	*p*-value
Sex (%)	Female	1570 (34.41)	683 (34.99)	0.671
	Male	2993 (65.59)	1269 (65.01)	
Age (years)		49.00 [40.00, 56.00]	50.00 [41.00, 56.00]	0.644
BMI (kg/m^2^)		23.67 [21.64, 25.68]	23.75 [21.75, 25.79]	0.156
SBP (mmHg)		123.00 [112.00, 134.00]	123.00 [113.00, 134.00]	0.601
DBP (mmHg)		75.00 [68.00, 83.00]	75.00 [68.00, 83.00]	0.769
History of hypertension (%)	Yes/	369 (8.09)	173 (8.86)	0.322
	No	4194 (91.91)	1779 (91.14)	
History of DM (%)	Yes	91 (1.99)	50 (2.56)	0.178
	No	4472 (98.01)	1902(97.44)	
History of stroke (%)	Yes	6 (0.13)	1 (0.05)	0.622
	No	4557 (99.87)	1951(99.95)	
Albumin (g/L)		45.20 [43.60, 46.90]	45.20 [43.60, 46.80]	0.446
Scr (μmol/L)		83.20 [72.55, 91.75]	82.95 [72.30, 91.50]	0.782
UA (μmol/L)		352.00 [293.00, 409.00]	350.00 [293.00, 409.00]	0.999
TG (mmol/L)		1.34 [0.95, 1.94]	1.33 [0.94, 1.95]	0.705
HDL-C (mmol/L)		1.29 [1.11, 1.50]	1.28 [1.11, 1.51]	0.719
LDL-C (mmol/L)		2.93 [2.43, 3.45]	2.96 [2.46, 3.49]	0.216
HbA1c (%)		5.30 [5.10, 5.60]	5.30 [5.10, 5.60]	0.377
Hb (g/L)		148.00 [137.00, 158.00]	148.00 [136.00, 157.00]	0.544

BMI: body mass index; DBP: diastolic blood pressure; DM: diabetes mellitus; Hb: hemoglobin; HbA1c: glycated hemoglobin A1c; HDL-C: high-density lipoprotein cholesterol; LDL-C: low-density lipoprotein cholesterol; SBP: systolic blood pressure; Scr: serum creatinine; TG: triglyceride; UA: uric acid.

### Predictor selection and identification

Fifteen variables were included in the univariate analysis (Table 2). The univariate analysis showed that patients with CKD exhibited a higher likelihood of being older; a greater prevalence of hypertension and DM; higher body mass index, systolic blood pressure, diastolic blood pressure, and serum creatinine, uric acid, triglyceride, and HbA1c levels; and lower serum albumin levels than those seen in healthy controls (all *p* < 0.05). Conversely, no statistically significant differences in sex, hemoglobin, high-density lipoprotein cholesterol, or low-density lipoprotein cholesterol levels were observed between the two groups (all *p* > 0.05).

**Table 2.** Univariate analysis of predictive factors for chronic kidney disease.

Variables		CKD		*p*-value
		No	Yes	
Sex (%)	Male	2917 (65.62)	76 (64.41)	0.860
	Female	1528 (34.38)	42 (35.59)	
History of hypertension (%)	No	4103 (92.31)	91 (77.12)	<0.001*
	Yes	342 (7.69)	27 (22.88)	
History of DM (%)	No	4370 (98.31)	102 (86.44)	<0.001*
	Yes	75 (1.69)	16 (13.56)	
History of stroke (%)	No	4439 (99.87)	118 (100.00)	>0.999
	Yes	6 (0.13)	0 (0.00)	
Age (years)^#^		49.00 [40.00, 55.00]	64.00 [57.25, 70.75]	<0.001*
BMI (kg/m^2^)^#^		23.61 [21.62, 25.64]	24.89 [22.80, 27.01]	<0.001*
SBP (mmHg)^#^		123.00 [112.00, 134.00]	134.00 [122.00, 146.50]	<0.001*
DBP (mmHg)^#^		75.00 [67.00, 83.00]	79.00 [71.25, 86.00]	<0.001*
Albumin (g/L)^#^		45.30 [43.60, 46.90]	44.80 [43.12, 46.18]	0.010*
Scr (μmol/L)^#^		83.00 [72.40, 91.50]	95.15 [79.38, 102.90]	<0.001*
UA (μmol/L)^#^		351.00 [292.00, 409.00]	373.50 [326.25, 432.75]	0.002*
TG (mmol/L)^#^		1.33 [0.95, 1.93]	1.67 [1.23, 2.47]	<0.001*
HDL-C (mmol/L)^#^		1.29 [1.11, 1.50]	1.31 [1.07, 1.47]	0.408
LDL-C (mmol/L)^#^		2.94 [2.43, 3.45]	2.88 [2.29, 3.47]	0.459
HbA1c (%)^#^		5.30 [5.00, 5.60]	5.60 [5.30, 6.07]	<0.001*
Hb (g/L)^#^		148.00 [137.00, 158.00]	143.00 [135.25, 155.00]	0.060

^#^ non-normal data distribution.

* *p* < 0.05.

BMI: body mass index; CKD: chronic kidney disease; DBP: diastolic blood pressure; DM: diabetes mellitus; Hb: hemoglobin; HbA1c: glycated hemoglobin A1c; HDL-C: high-density lipoprotein cholesterol; LDL-C: low-density lipoprotein cholesterol; SBP: systolic blood pressure; Scr: serum creatinine; TG: triglyceride; UA: uric acid.

To explore potential multicollinearity as a result of correlations between independent variables, we conducted collinearity analyses for all significant variables in the univariate analysis before performing multivariate logistic regression (Table 3). As demonstrated in Table 3, the maximum value of the variance inflation factor was less than three and the minimum value of tolerance was greater than 0.3, indicating no serious multicollinearity between predictors.

**Table 3.** The tolerance and variance inflation factor for the training cohort.

Variables	Tolerance	VIF
Age	0.703	1.422
BMI	0.718	1.393
SBP	0.358	2.792
DBP	0.372	2.685
History of hypertension	0.822	1.216
History of DM	0.843	1.186
Albumin	0.837	1.195
Scr	0.650	1.538
UA	0.573	1.746
TG	0.821	1.218
HbA1c	0.779	1.284

BMI: body mass index; DBP: diastolic blood pressure; DM: diabetes mellitus; HbA1c: glycated hemoglobin A1c; SBP: systolic blood pressure; Scr: serum creatinine; TG: triglyceride; UA: uric acid; VIF: variance inflation factor.

Because no substantial multicollinearity was observed, all variables were included in the multivariate logistic regression model (Table 4). The results indicated that age (odds ratio [OR] 1.118, 95% confidence interval [CI] 1.090–1.145, *p* < 0.001), history of DM (OR 3.683, 95% CI 1.791–7.575, *p* < 0.001), and serum creatinine (OR 1.068, 95% CI 1.048–1.088, *p* < 0.001) and triglyceride levels (OR 1.201, 95% CI 1.083–1.332, *p* = 0.001 < 0.05) were independent predictors for CKD in the healthy population.

**Table 4.** Multivariate logistic regression analysis of predictive factors for chronic kidney disease.

Variables	β	SE	Wald	*p*-value	OR	OR 95%CI	
						L	U
History of hypertension	0.336	0.269	1.563	0.211	1.399	0.826	2.369
History of DM	−1.304	0.368	12.556	<0.001*	0.272	0.132	0.558
Age	0.111	0.013	78.905	<0.001*	1.118	1.090	1.145
BMI	0.036	0.039	0.852	0.356	1.037	0.961	1.119
SBP	0.015	0.008	3.36	0.067	1.015	0.999	1.032
DBP	−0.011	0.014	0.614	0.433	0.989	0.963	1.016
HbA1c	0.119	0.106	1.264	0.261	1.126	0.916	1.385
Albumin	−0.066	0.044	2.175	0.140	0.937	0.858	1.022
Scr	0.065	0.01	47.048	<0.001*	1.068	1.048	1.088
UA	0.000	0.002	0.000	0.988	1.000	0.997	1.003
TG	0.183	0.053	11.965	0.001*	1.201	1.083	1.332

* *p* < 0.05.

BMI: body mass index; CI: confidence interval; DBP: diastolic blood pressure; DM: diabetes mellitus; HbA1c: glycated hemoglobin A1c; L: lower limit; OR: odds ratio; SBP: systolic blood pressure; Scr: serum creatinine; SE: standard error; TG: triglyceride; U: upper limit; UA: uric acid.

### Model construction and internal validation

**Figure 1.**
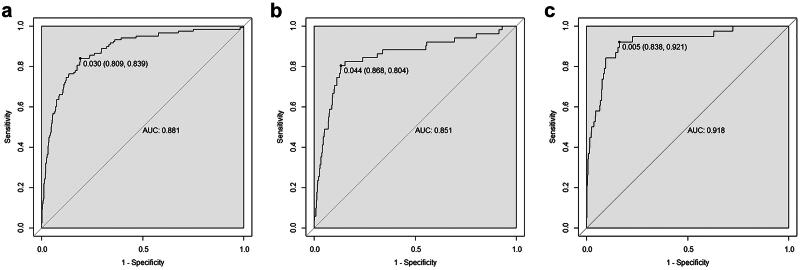
ROC curves. (a) ROC curves in the training cohort; (b) ROC curves in the internal validation cohort; (c) ROC curves in the external validation cohort. AUC: area under the curve; ROC: receiver operating characteristic.

**Figure 2.**
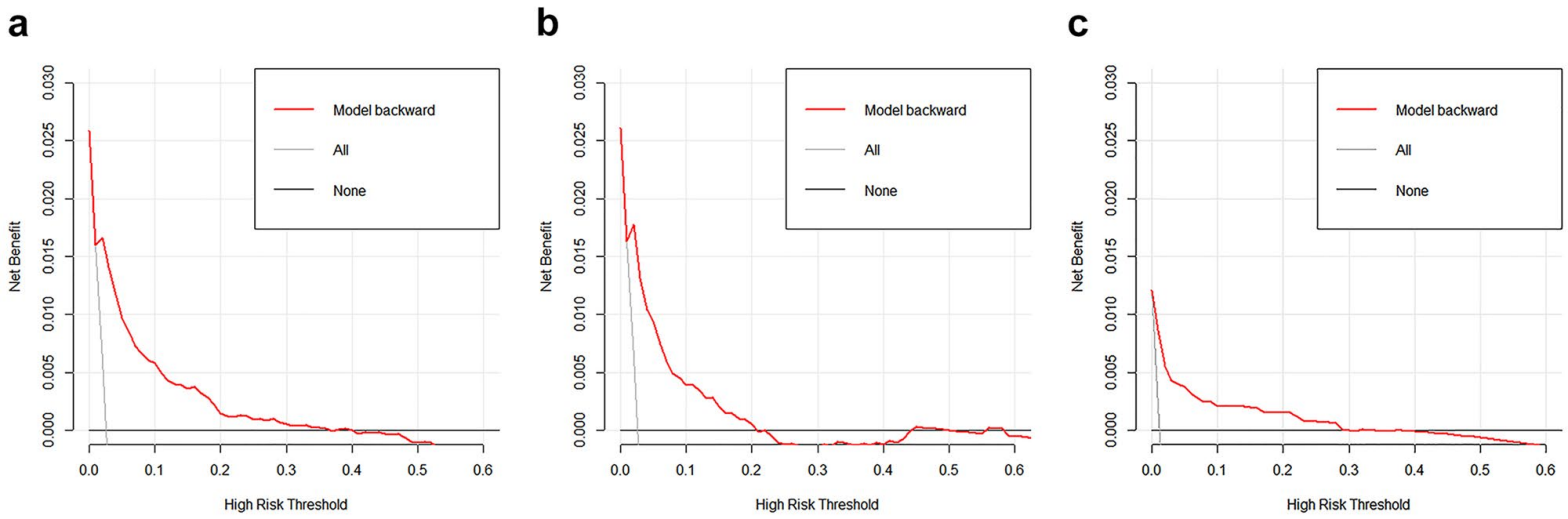
Decision curve analyses. (a) Decision curve analyses in the training cohort; (b) decision curve analyses in the internal validation cohort; (c) decision curve analyses in the external validation cohort.

The backward stepwise AIC procedure was performed by iteratively adding and removing model predictors to identify the subset with the best performance (lowest AIC score) [13]. The final predictive model included six variables: age, history of DM, systolic blood pressure, and serum creatinine, albumin, and triglyceride levels (AIC = 833.8563). The model exhibited satisfactory prediction accuracy for both the training and internal validation datasets (training dataset, AUC 0.8806, 95% CI 0.8472–0.9141, Figure 1a; internal validation dataset, AUC 0.8506, 95% CI 0.7856–0.9156, Figure 1b). The calibration plot demonstrated that the model was appropriate for both the training and internal validation datasets (Appendix 4, supplementary material). The DCA also demonstrated satisfactory clinical practicability (Figure 2).

**Figure 3.**
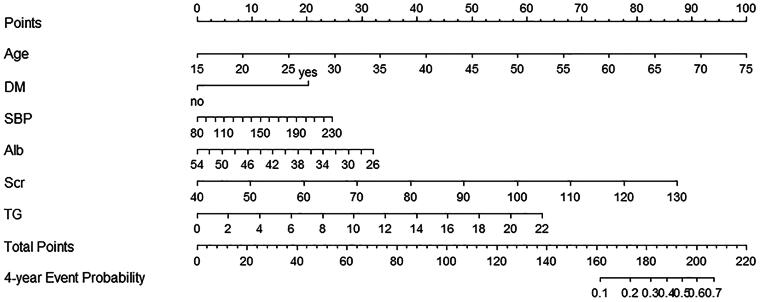
Nomogram for prediction of risk of developing CKD within 4 years in the healthy population. To use the nomogram, draw an upward vertical line from each variable axis to the ‘Points’ bar to calculate the corresponding points for each variable. Then sum the covariate points and draw a downward vertical line from the ‘Total Points’ bar to obtain the predictive probability of developing CKD within 4 years. The higher the total points, the higher the risk of CKD. Alb: albumin; CKD: chronic kidney disease; DM: diabetes mellitus; SBP: systolic blood pressure; Scr: serum creatinine; TG: serum triglyceride.

A nomogram was created using the model results (Figure 3). Older age; history of DM; higher systolic blood pressure and serum creatinine and triglyceride levels; and lower serum albumin levels were associated with an increased risk of CKD (Figure 3). In addition, the length of the line in the nomogram directly correlated with the likelihood of a factor contributing to the development of CKD. Consequently, we can conclude that age had the greatest impact on the risk of CKD, whereas a history of DM had the least impact.

An example of score calculation using the nomogram is provided in Appendix 5, supplementary material. If a 75-year-old healthy individual (100 points) had a known history of DM (20 points), systolic blood pressure of 140 mmHg (10 points), serum albumin level of 40 g/L (16 points), serum creatinine level of 90 μmol/L (48 points), and triglyceride level of 4 mmol/L (12 points), the total points added up to 206, and the corresponding predictive probability of developing CKD within 4 years was approximately 69%.

**Figure 4.**
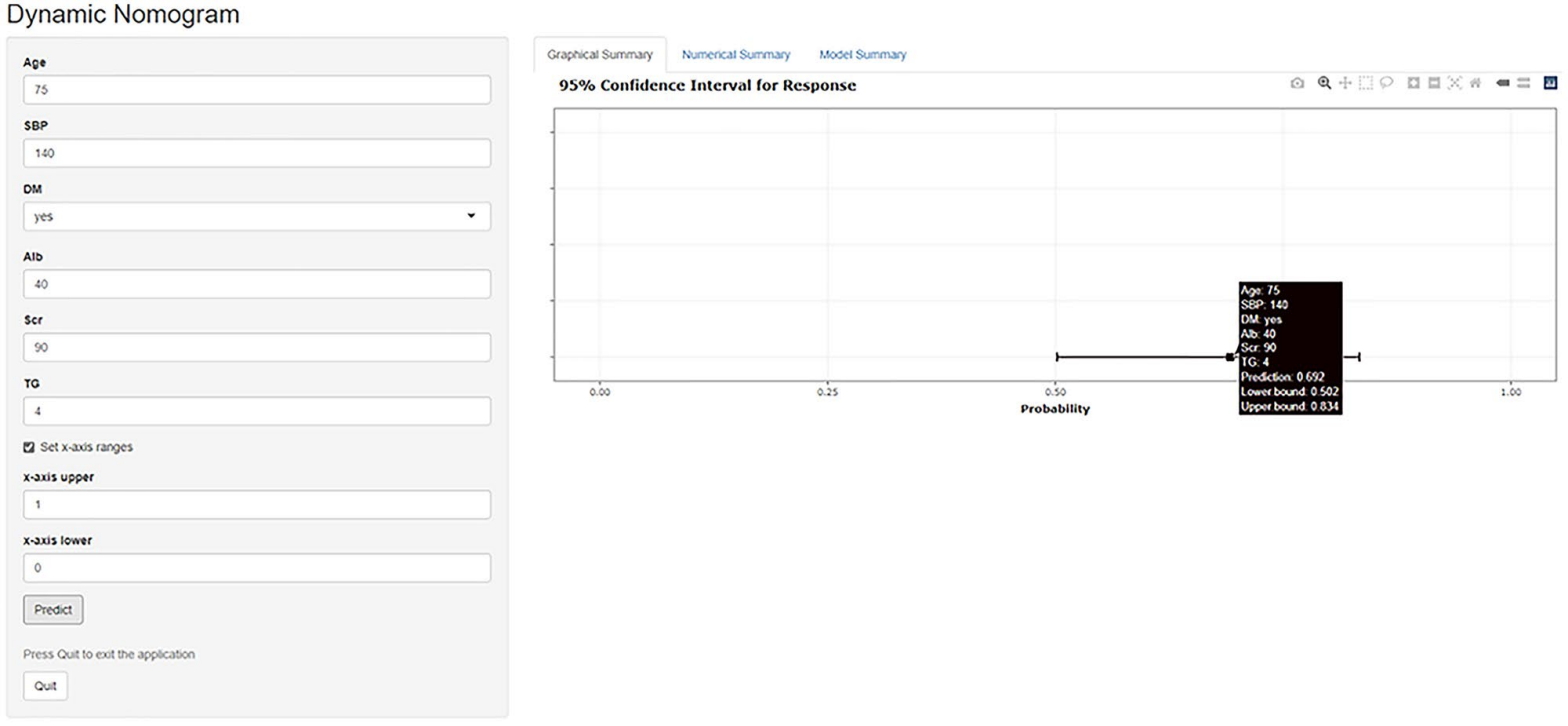
The intuitive interface of the web-based nomogram (https://luochuxuan.shinyapps.io/dynnomapp/). Instruction for using the web-based nomogram: Enter model parameter values in the left input box and click the ‘Predict’ button below to start the analysis. The corresponding individual probability of developing CKD within 4 years can be queried in the ‘Graphical Summary’ and ‘Numerical Summary’ modules on the right side of the interface. CKD: chronic kidney disease; Alb: albumin; DM: diabetes mellitus; SBP: systolic blood pressure; Scr: serum creatinine; TG: serum triglyceride.

A web-based nomogram was developed to enhance the accessibility and usability for physicians and individuals interested in healthcare. Figure 4 shows a screenshot of this nomogram, which can be freely accessed at https://luochuxuan.shinyapps.io/dynnomapp/.

### External model validation

To further substantiate the performance and robustness of the model, we employed an autonomous cohort for external validation. The model demonstrated exceptional discriminatory prowess, as evidenced by an AUC of 0.9183 (95% CI 0.8698–0.9669) in the external validation set (Figure 1c). The DCA outcomes in the external validation cohort also exhibited consistent stability (Figure 2c).

## Discussion

We successfully built and validated a CKD risk prediction nomogram model using relatively large, multicenter datasets from China. Nomograms are graphical representations of sophisticated mathematical models [14] and have been extensively employed as prognostic tools in clinical oncology [15–18]. However, their use in nephrology is relatively limited, despite the gradual increase in the number of applications [19]. In contrast to previous nomograms, which were only capable of calculating an approximate value, this nomogram offers an accurate result. As an alternative to the traditional nomogram, we created a nomogram that can be operated on a website to predict the probability of developing CKD within 4 years in a healthy individual.

Numerous scholars both domestically and internationally have conducted research on predicting the risk of CKD, highlighting it as a current focal point in research [20,21]. In 2010, Dr. Chien and his research team at National Taiwan University developed the initial CKD risk prediction model in China utilizing clinical data from 5168 individuals without preexisting kidney conditions [20]. The clinical model was designed to enhance the management and mitigation of CKD by incorporating factors such as age, body mass index, diastolic blood pressure, history of type 2 diabetes, and history of stroke. Subsequently, a biochemical model was devised, incorporating variables such as age, diastolic blood pressure, history of stroke, uric acid, postprandial blood glucose, HbA1c, and proteinuria (>100mg/dl). The AUC values for the clinical and biochemical models were 0.768 and 0.765, respectively. While this models demonstrate some predictive capability for the 4-year incidence of CKD, further enhancements are necessary to improve its predictive efficiency. Furthermore, in 2019, another CKD risk prediction model was developed by Dr. Nelson’s research team at the National Institutes of Health [21]. This model was based on the analysis of individual data from approximately 800,000 individuals with diabetes and nearly 4.4 million non-diabetic adults across 28 countries. The models for non-diabetic individuals incorporated variables such as age, sex, race, body mass index, history of hypertension, history of cardiovascular disease, smoking habits, eGFR, and proteinuria. Building upon this model, the model for diabetic patients introduced two additional variables: HbA1c and the utilization of insulin therapy. These models effectively forecasted the 5-year risk of CKD. Regrettably, our model is unable to directly assess its predictive efficacy in comparison to the aforementioned models. This limitation arises from the constraints of cost and time, which restricted data collection to the period spanning from January 2017 to December 2021. Consequently, our predictive models were developed solely for forecasting CKD risk over a 4-year timeframe. Future research endeavors will seek to construct a 5-year CKD risk prediction model tailored specifically for Chinese residents, enabling a comparative analysis with existing models.

With respect to GFR estimation, which is distinct from other studies [10], our study employed the CKD-EPI equation to estimate the eGFR instead of the Modification of Diet in Renal Disease (MDRD) equation. The MDRD formula was originally devised in 1999 by Levey et al. [22]. Although the MDRD method is endorsed for routine clinical use by the K/DOQI and Kidney Disease Improving Global Outcome (KDIGO) guidelines [1,23,24], it has been reported to underestimate the actual GFR in populations with normal renal function [25,26]. A previous study showed that the CKD-EPI formula exhibited a higher level of accuracy than the MDRD formula, especially in individuals with an eGFR exceeding 60 mL/min/1.73 m^2^ [9]. Moreover, the diagnosis of CKD in our study was based solely on eGFR, without considering proteinuria as a defining criterion. It is crucial to recognize that albuminuria and decreased eGFR can be influenced by different factors. For instance, proteinuria is an early indicator of diabetic nephropathy, while eGFR may remain within normal limits. Additionally, individuals with metabolic syndrome may develop proteinuria while maintaining normal renal function. Therefore, our CKD definition did not include the presence of proteinuria, considering these aspects. Moving forward, our goal is to develop a predictive model for albuminuria and evaluate its predictive accuracy compared to our current model.

The incidence rate of CKD within a 4-year-period in this study was 2.37% (229/9667), which was lower than that reported in Taiwan (3.68%) [20]. We consider that several factors could contribute to this outcome, including sample size, population demographics, geographical environment, and dietary habits of the participants. In addition, CKD screening using different approaches may affect the incidence rate. Regular follow-up every 3 months is recommended after the diagnosis of CKD is established, based on our clinical experience.

Six covariates, namely, age, history of DM, systolic blood pressure, and serum creatinine, albumin, and triglyceride levels, were included in the final nomogram model as predictors. Age was identified as an independent predictor of CKD, which is consistent with the characteristics of kidney disease [27]. Approximately half of the individuals aged >75 years met the diagnostic criteria for CKD according to the 2012 KDIGO guidelines [28]. This may be explained by the decreased renal function in the elderly population. Over the age of 40 years, GFR is estimated to decline by approximately 1 mL/min/year and accelerate slightly later in life [29]. The association between a history of DM, systolic blood pressure, and the risk of CKD onset has been corroborated by other studies [30], indicating the significance of blood pressure management in hypertensive patients and glycemic control in diabetic patients to prevent CKD development. Serum creatinine levels are frequently used to evaluate renal function, as impaired kidney function may lead to incomplete excretion of creatinine, consequently elevating the creatinine concentration in the bloodstream [31]. Serum albumin levels have been extensively employed as a dependable nutritional indicator for monitoring nutritional deficiencies [32]. Recently, Alves et al. suggested that patients with hypoalbuminemia have a higher risk of developing CKD, which is consistent with the findings of the present investigation [33]. Our study revealed a positive correlation between elevated triglyceride levels and risk of CKD. This may be because hypertriglyceridemia often presents with chronic comorbid conditions such as abdominal obesity, hypertension, and DM.

Despite the advantages of this study, certain limitations exist. First, as the multicenter data were limited to third-grade hospitals in a single city in China, the findings of this research cannot be directly extrapolated to the rest of the world. Hence, external validation using data from other countries or populations will be required in the future. Second, it is important to note that this study was retrospective in nature, and a proportion of participants was lost to follow-up, which may have caused a degree of selection bias. Further large cohort prospective studies are warranted to validate these findings. Third, since the study only included individuals who participated in annual physical exams for five consecutive years, it is possible that those unable to attend annual examinations due to developing diseases, including the anticipated outcome, might have been inadvertently excluded from the study. Fourth, the current study is limited by not examining the differences in patient characteristics before and after developing CKD. However, we intend to address this limitation in a future manuscript. Additionally, the lack of serum cystatin C level data in our physical examination dataset prevented us from examining its impact on renal function. Fifth, the definition of CKD employed in our study diverged from that outlined in the current guidelines. Specifically, given that the participants in this study were individuals undergoing annual routine medical examinations, we defined CKD as an eGFR of less than 60 mL/min/1.73m^2^ for 1 or more consecutive years, corresponding to the annual data collection interval. Consequently, this could potentially result in an underestimation of the true prevalence of patients with CKD. Despite the potential for underestimation, this approach facilitates a more precise diagnosis of CKD, thereby enhancing the credibility of the model. Lastly, the study’s reliance on individuals undergoing physical examinations, without access to recent medication usage information, limits our capacity to assess the effects of medications on the development and progression of CKD. As such, these limitations may somewhat restrict the generalizability of our findings.

This study identified age, history of DM, and serum creatinine and triglyceride levels as significant independent predictors of new incident CKD within a 4-year period in a population receiving routine physical examinations. Additionally, a nomogram was constructed using six predictors, namely age, history of DM, systolic blood pressure, serum albumin, creatinine, and triglyceride levels, to facilitate the early prediction of CKD. The accuracy of the nomogram was confirmed through rigorous internal and external validations, consistently demonstrating its robust predictive capability. These findings may help in identifying individuals at heightened risk of developing CKD, thus facilitating early detection, prevention, and intervention strategies that may contribute to a reduction in the occurrence of CKD.

## Supplementary Material

Figure 1.jpg

Figure 2.jpg

Figure 4.jpg

Appendix 5.docx

Appendix 3.docx

Appendix 2.docx

Appendix 1.docx

Appendix 4.docx

Figure 3.jpg

## Data Availability

The datasets of this study are available from the corresponding author on reasonable request.
