# Supplementary figures and images for: Development, validation, and visualization of a web-based nomogram for predicting chronic kidney disease incidence at health examination centers
Source: Ren Fail. 2024 Oct 8;46(2):2398183. doi: 10.1080/0886022X.2024.2398183 (PMC11463019; doi:10.1080/0886022X.2024.2398183)

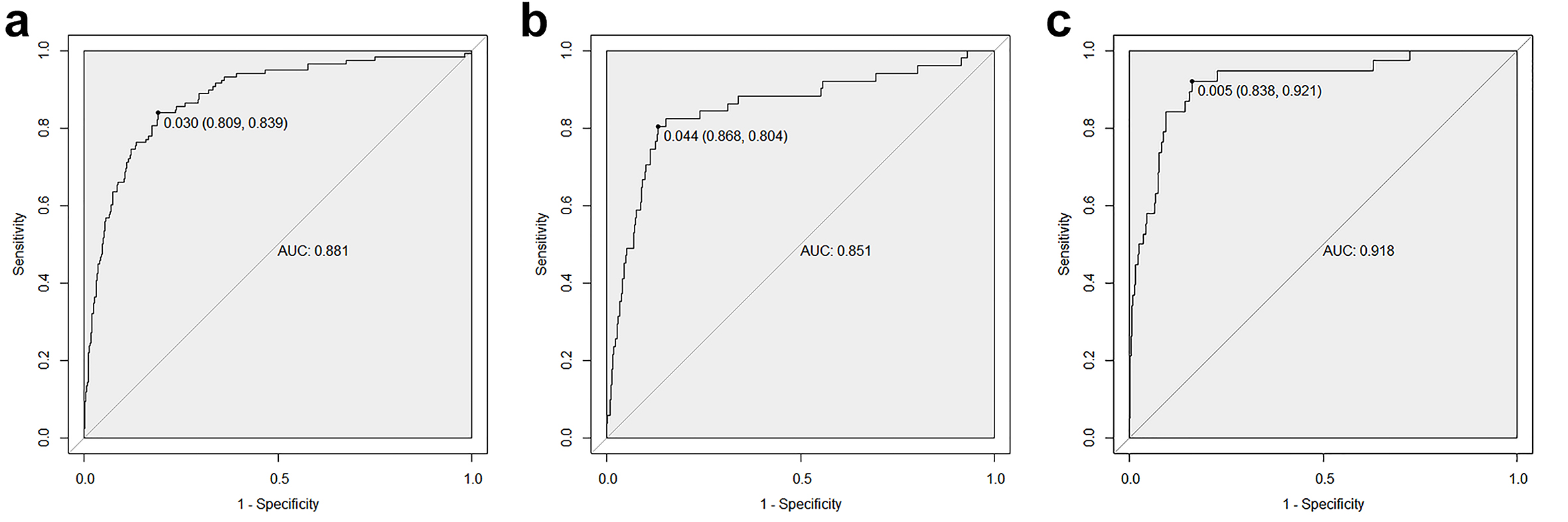

Supplement: Figure 1.jpg [file IRNF_A_2398183_SM3867.jpg]

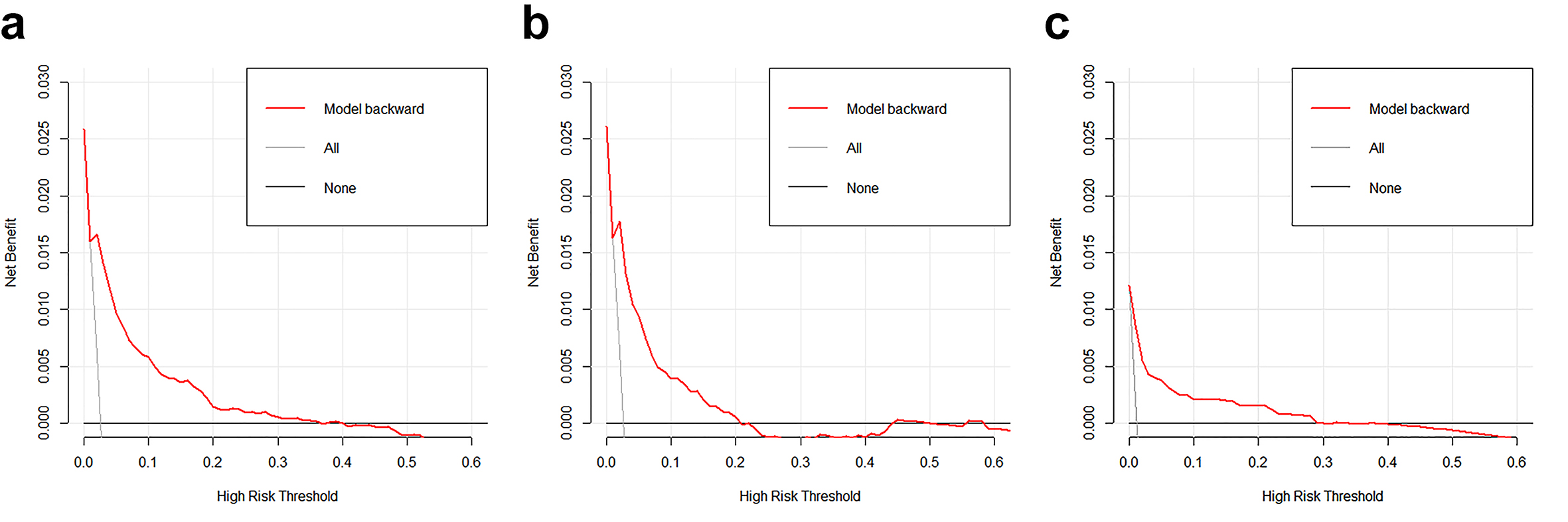

Supplement: Figure 2.jpg [file IRNF_A_2398183_SM3866.jpg]

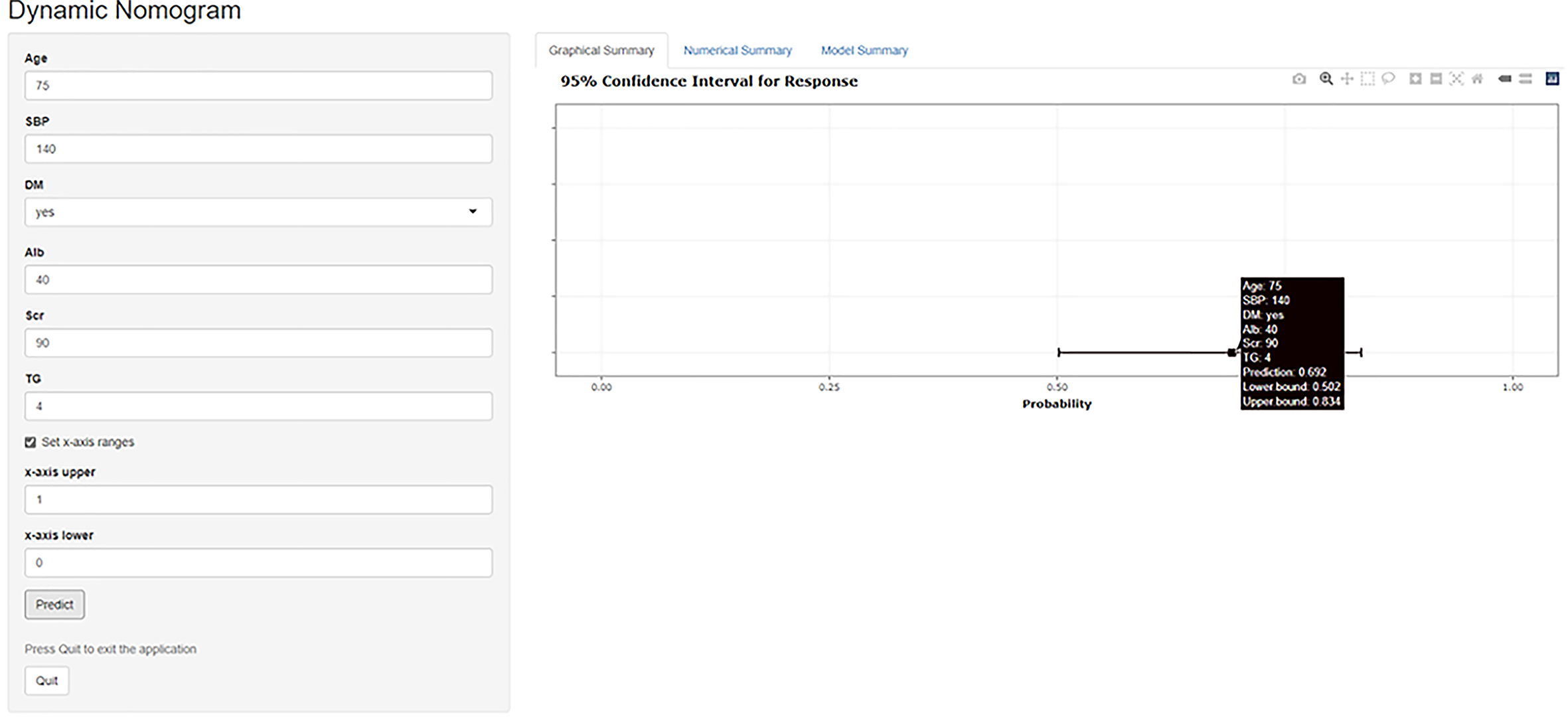

Supplement: Figure 4.jpg [file IRNF_A_2398183_SM3865.jpg]

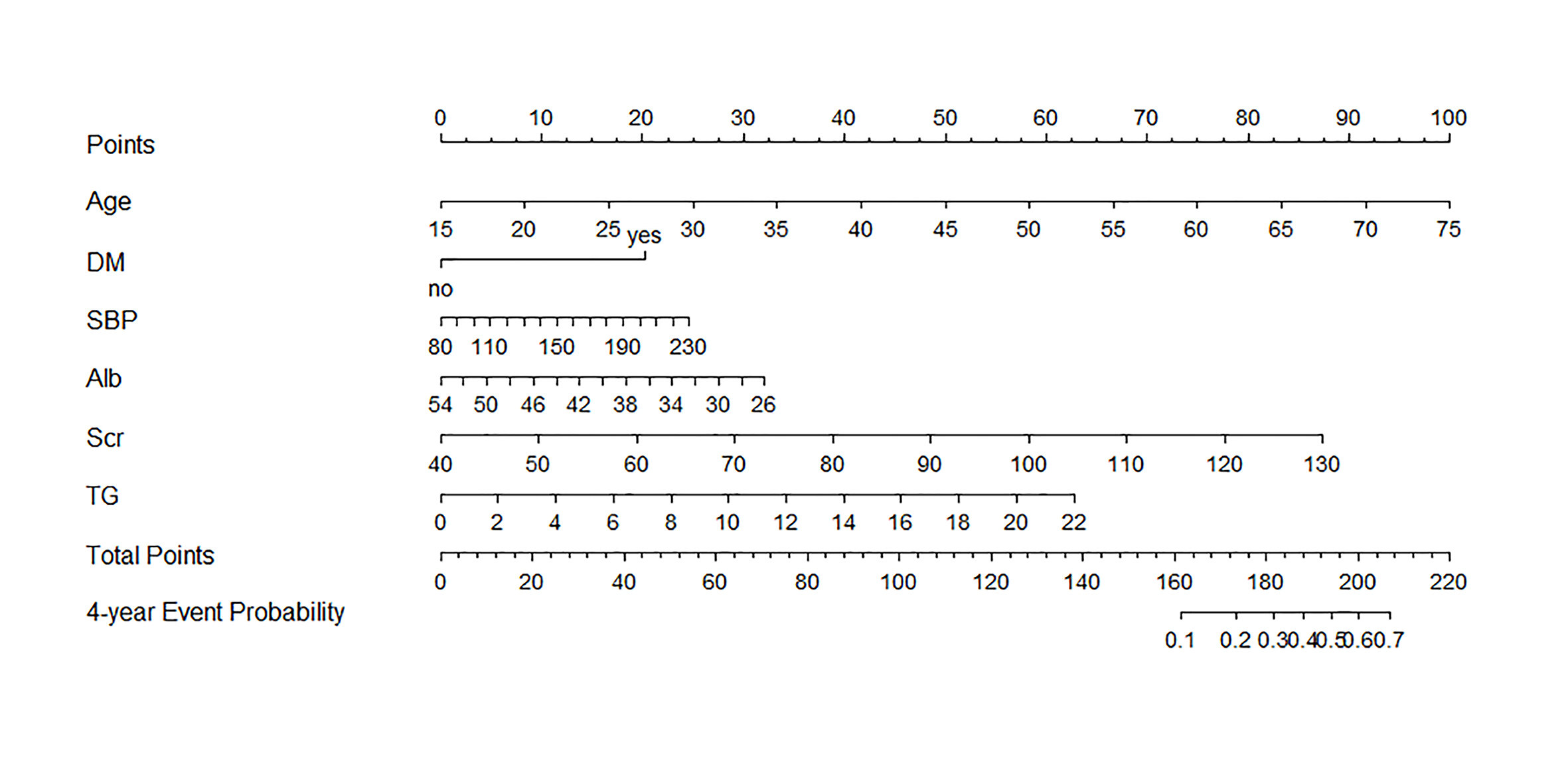

Supplement: Figure 3.jpg [file IRNF_A_2398183_SM3859.jpg]
