# Supplementary material for: Development, validation, and visualization of a web-based nomogram for predicting chronic kidney disease incidence at health examination centers
Source: Ren Fail. 2024 Oct 8;46(2):2398183. doi: 10.1080/0886022X.2024.2398183 (PMC11463019; doi:10.1080/0886022X.2024.2398183)
Supplement: Appendix 5.docx [file IRNF_A_2398183_SM3864.docx]

**
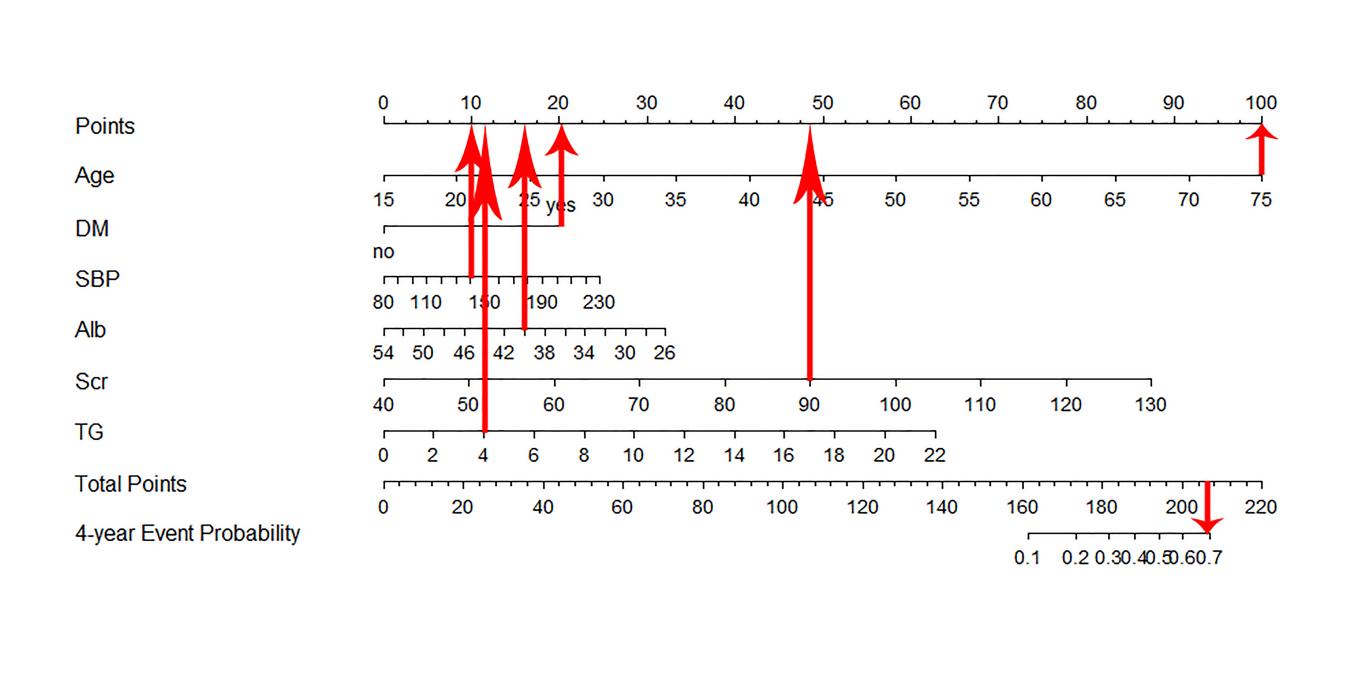
**

**Supplementary Fig. 3:** An example of how to use the nomogram. The use of the nomogram is exemplified with a hypothetical 75-year-old healthy individual (100 points) with a previous history of diabetes mellitus (20 points), a systolic blood pressure of 140 mmHg (10 points), a serum albumin level of 40 g/L (16 points), a serum creatinine level of 90 μmol/L (48 points) and a triglyceride level of 4 mmol/L (12 points). Based on the total points (206 points), we can estimate that this individual has approximately 0.69 risk of developing CKD within 4 years (shown in the nomogram). Abbreviations: Alb, albumin; CKD, chronic kidney disease; DM, diabetes mellitus; SBP, systolic blood pressure; Scr, serum creatinine; TG, serum triglyceride.
