# Supplementary material for: Development, validation, and visualization of a web-based nomogram for predicting chronic kidney disease incidence at health examination centers
Source: Ren Fail. 2024 Oct 8;46(2):2398183. doi: 10.1080/0886022X.2024.2398183 (PMC11463019; doi:10.1080/0886022X.2024.2398183)
Supplement: Appendix 3.docx [file IRNF_A_2398183_SM3863.docx]

**Supplementary Table 2.** Comparison of baseline characteristics between internal validation cohort and external validation cohort

| **Variables** | | **Internal validation cohort (n=1952)** | **External validation cohort (n=3152)** | ***p*-value** |
| --- | --- | --- | --- | --- |
| Sex (%) | Female | 683 (34.99) | 1339 (42.48) | <0.001 |
|  | Male | 1269 (65.01) | 1813 (57.52) |  |
| Age (years) | | 50.00 [41.00, 56.00] | 36.00 [29.00, 48.00] | <0.001 |
| BMI (kg/m^2^) | | 23.75 [21.75, 25.79] | 23.23 [20.84, 25.61] | <0.001 |
| SBP (mmHg) | | 123.00 [113.00, 134.00] | 117.00 [107.00, 129.00] | <0.001 |
| DBP (mmHg) | | 75.00 [68.00, 83.00] | 70.00 [63.00, 77.00] | <0.001 |
| History of hypertension (%) | Yes | 173 (8.86) | 308 (9.77) | 0.280 |
|  | No | 1779 (91.14) | 2844 (90.23) |  |
| History of DM (%) | Yes | 50 (2.56) | 146 (4.63) | <0.001 |
|  | No | 1902(97.44) | 3006 (95.37) |  |
| History of stroke (%) | Yes | 1 (0.05) | / | / |
|  | No | 1951(99.95) | / |  |
| Albumin (g/L) | | 45.20 [43.60, 46.80] | 47.90 [46.40, 49.50] | <0.001 |
| Scr (μmol/L) | | 82.95 [72.30, 91.50] | 64.20 [54.00, 73.70] | <0.001 |
| UA (μmol/L) | | 350.00 [293.00, 409.00] | 327.50 [270.20, 387.98] | <0.001 |
| TG (mmol/L) | | 1.33 [0.94, 1.95] | 1.10 [0.77, 1.64] | <0.001 |
| HDL-C (mmol/L) | | 1.28 [1.11, 1.51] | 1.20 [1.05, 1.40] | <0.001 |
| LDL-C (mmol/L) | | 2.96 [2.46, 3.49] | 2.48 [2.06, 3.00] | <0.001 |
| HbA1c (%) | | 5.30 [5.10, 5.60] | 5.30 [5.10, 5.40] | <0.001 |
| Hb (g/L) | | 148.00 [136.00, 157.00] | 149.00 [136.00, 159.00] | 0.105 |

BMI: body mass index; DBP: diastolic blood pressure; DM: diabetes mellitus; Hb: hemoglobin; HbA1c: glycated hemoglobin A1c; HDL-C: high-density lipoprotein cholesterol; LDL-C: low-density lipoprotein cholesterol; SBP: systolic blood pressure; Scr: serum creatinine; TG: triglyceride; UA: uric acid.
