# Supplementary material for: Development, validation, and visualization of a web-based nomogram for predicting chronic kidney disease incidence at health examination centers
Source: Ren Fail. 2024 Oct 8;46(2):2398183. doi: 10.1080/0886022X.2024.2398183 (PMC11463019; doi:10.1080/0886022X.2024.2398183)
Supplement: Appendix 2.docx [file IRNF_A_2398183_SM3862.docx]

**Supplementary Table 1.** Comparison of baseline characteristics between training cohort and external validation cohort

| **Variables** | | **Training cohort (n=4563)** | **External validation cohort (n=3152)** | ***p*-value** |
| --- | --- | --- | --- | --- |
| Sex (%) | Female | 1570 (34.41) | 1339 (42.48) | <0.001 |
|  | Male | 2993 (65.59) | 1813 (57.52) |  |
| Age (years) | | 49.00 [40.00, 56.00] | 36.00 [29.00, 48.00] | <0.001 |
| BMI (kg/m^2^) | | 23.67 [21.64, 25.68] | 23.23 [20.84, 25.61] | <0.001 |
| SBP (mmHg) | | 123.00 [112.00, 134.00] | 117.00 [107.00, 129.00] | <0.001 |
| DBP (mmHg) | | 75.00 [68.00, 83.00] | 70.00 [63.00, 77.00] | <0.001 |
| History of hypertension (%) | Yes | 369 (8.09) | 308 (9.77) | 0.010 |
|  | No | 4194 (91.91) | 2844 (90.23) |  |
| History of DM (%) | Yes | 91 (1.99) | 146 (4.63) | <0.001 |
|  | No | 4472 (98.01) | 3006 (95.37) |  |
| History of stroke (%) | Yes | 6 (0.13) | / | / |
|  | No | 4557(99.87) | / |  |
| Albumin (g/L) | | 45.20 [43.60, 46.90] | 47.90 [46.40, 49.50] | <0.001 |
| Scr (μmol/L) | | 83.20 [72.55, 91.75] | 64.20 [54.00, 73.70] | <0.001 |
| UA (μmol/L) | | 352.00 [293.00, 409.00] | 327.50 [270.20, 387.98] | <0.001 |
| TG (mmol/L) | | 1.34 [0.95, 1.94] | 1.10 [0.77, 1.64] | <0.001 |
| HDL-C (mmol/L) | | 1.29 [1.11, 1.50] | 1.20 [1.05, 1.40] | <0.001 |
| LDL-C (mmol/L) | | 2.93 [2.43, 3.45] | 2.48 [2.06, 3.00] | <0.001 |
| HbA1c (%) | | 5.30 [5.10, 5.60] | 5.30 [5.10, 5.40] | <0.001 |
| Hb (g/L) | | 148.00 [137.00, 158.00] | 149.00 [136.00, 159.00] | 0.159 |

BMI: body mass index; DBP: diastolic blood pressure; DM: diabetes mellitus; Hb: hemoglobin; HbA1c: glycated hemoglobin A1c; HDL-C: high-density lipoprotein cholesterol; LDL-C: low-density lipoprotein cholesterol; SBP: systolic blood pressure; Scr: serum creatinine; TG: triglyceride; UA: uric acid.
