# Supplementary material for: Development, validation, and visualization of a web-based nomogram for predicting chronic kidney disease incidence at health examination centers
Source: Ren Fail. 2024 Oct 8;46(2):2398183. doi: 10.1080/0886022X.2024.2398183 (PMC11463019; doi:10.1080/0886022X.2024.2398183)
Supplement: Appendix 1.docx [file IRNF_A_2398183_SM3861.docx]

**
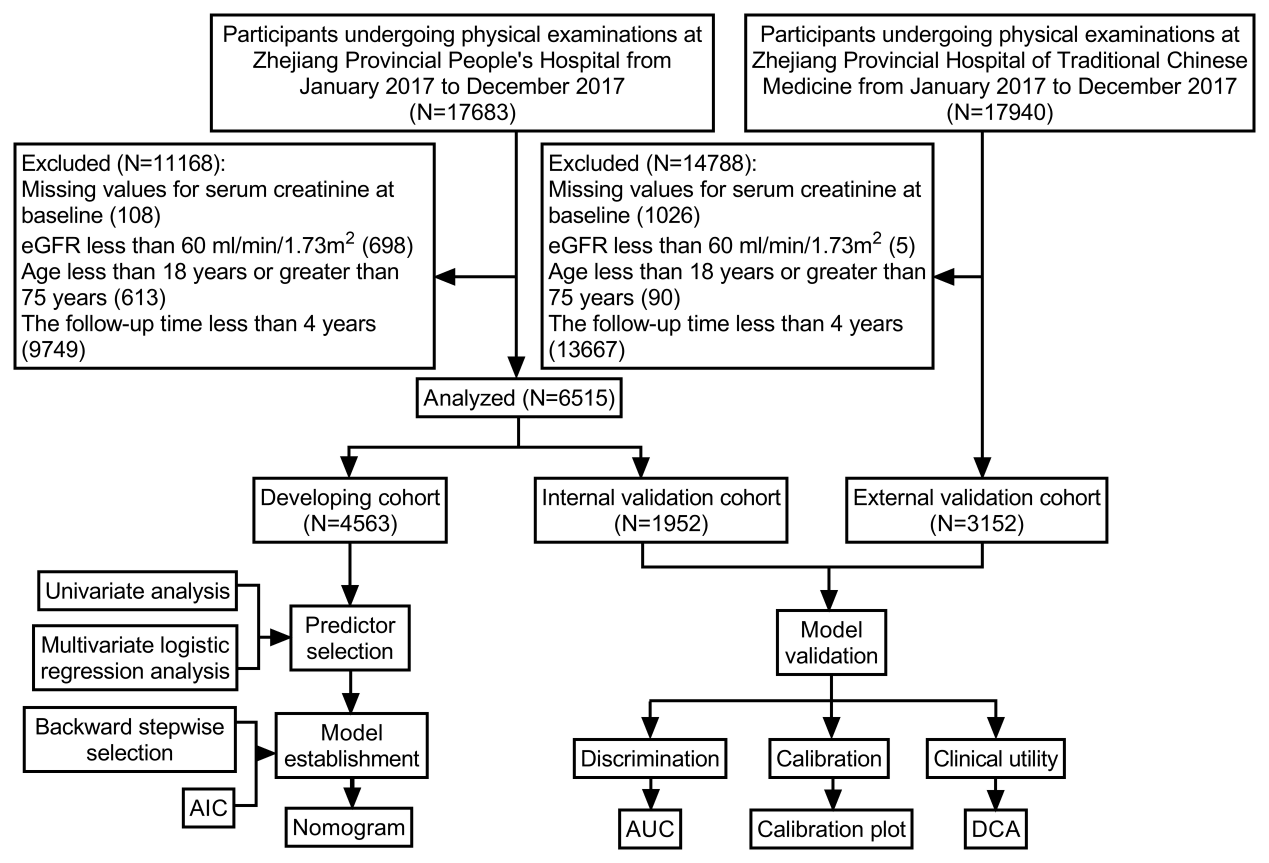
Supplementary Fig. 1:** Study flowchart. Abbreviations: AIC, Akaike information criterion; AUC, area under the curve; DCA, decision curve analysis; eGFR, estimated glomerular filtration rate.
