# Supplementary material for: Development, validation, and visualization of a web-based nomogram for predicting chronic kidney disease incidence at health examination centers
Source: Ren Fail. 2024 Oct 8;46(2):2398183. doi: 10.1080/0886022X.2024.2398183 (PMC11463019; doi:10.1080/0886022X.2024.2398183)
Supplement: Appendix 4.docx [file IRNF_A_2398183_SM3860.docx]

**
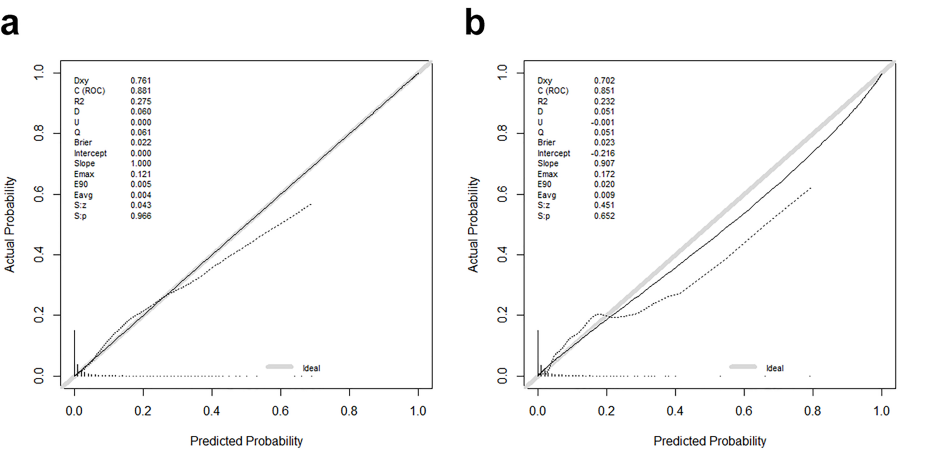
**

**Supplementary Fig. 2:** Calibration curves. (a) Calibration curves in the training cohort; (b) calibration curves in the internal validation cohort.
